# Machine Learning-Based Modeling and Generic Design Optimization Methodology for Radio-Frequency Microelectromechanical Devices

**DOI:** 10.3390/s23084001

**Published:** 2023-04-14

**Authors:** Rayan Bajwa, Murat Kaya Yapici

**Affiliations:** 1 Faculty of Engineering and Natural Sciences, Sabanci University, TR 34956 Istanbul, Turkey; rayanbajwa@sabanciuniv.edu; 2 Department of Electrical Engineering, University of Washington, Seattle, WA 98195, USA; 3 Sabanci University SUNUM Nanotechnology Research Center, TR 34956 Istanbul, Turkey

**Keywords:** 5G, cantilever, electromechanical RF optimization, finite element modeling, low actuation voltage, mechanical reliability, pull-in, RF-MEMS switch, shunt switch, stiction, switch design, von Mises stress

## Abstract

RF-MEMS technology has evolved significantly over the years, during which various attempts have been made to tailor such devices for extreme performance by leveraging novel designs and fabrication processes, as well as integrating unique materials; however, their design optimization aspect has remained less explored. In this work, we report a computationally efficient generic design optimization methodology for RF-MEMS passive devices based on multi-objective heuristic optimization techniques, which, to the best of our knowledge, stands out as the first approach offering applicability to different RF-MEMS passives, as opposed to being customized for a single, specific component. In order to comprehensively optimize the design, both electrical and mechanical aspects of RF-MEMS device design are modeled carefully, using coupled finite element analysis (FEA). The proposed approach first generates a dataset, efficiently spanning the entire design space, based on FEA models. By coupling this dataset with machine-learning-based regression tools, we then generate surrogate models describing the output behavior of an RF-MEMS device for a given set of input variables. Finally, the developed surrogate models are subjected to a genetic algorithm-based optimizer, in order to extract the optimized device parameters. The proposed approach is validated for two case studies including RF-MEMS inductors and electrostatic switches, in which the multiple design objectives are optimized simultaneously. Moreover, the degree of conflict among various design objectives of the selected devices is studied, and corresponding sets of optimal trade-offs (pareto fronts) are extracted successfully.

## 1. Introduction

Ever-growing demands for high-speed wireless communication systems have put a more stringent constraint on the performance of radio-frequency integrated circuits (RFICs), to keep up with the upcoming 5G standards. Therefore, along with better performing active circuitry, high-performance RF passive components have also become a key to realize any modern-day RFIC transceiver. Unfortunately, owing to inherent technological limitations, RF passive devices based on standard IC fabrication techniques face a large number of losses (e.g., substrate proximity loss), and hence restrict the overall performance of RFICs [1,2,3]. On the other hand, the field of MEMSs (microelectromechanical systems) has surfaced as a potential substitute for fabricating integrated high-performance on-chip RF passive components using advanced micromachining techniques [1,2,3,4]. MEMS-based RF passives (i.e., inductors, tunable capacitors, switches, transformers, phase shifters, etc.), also referred to as RF-MEMSs, exhibit high linearity, lower losses, high quality factors, and better power-handling abilities over larger bandwidths [2,5,6].

Nevertheless, the design of monolithic RF-MEMS passive components poses a primary technical challenge against the development of integrated RFICs. The coupled electromechanical nature of RF-MEMS components requires careful design and modeling of such devices in two different physical domains, thus formulating a typical multi-physics problem [7]. Hence, to achieve optimal performance metrics, a robust design optimization approach for RF-MEMS components is essential.

Generally, the design optimization techniques for RF-MEMS passive devices are based on intuitive approaches, in which designers focus on optimizing one or two of the most critical performance parameters (design objectives), while keeping others within pre-defined limits [8,9,10]. This approach, while time-efficient, does not lead to an optimal design, since it relies entirely on designers’ very own expertise and experience. Therefore, in order to alleviate these design challenges, a more advanced optimization approach must be followed. Although formal design optimization techniques exist and are quite common in the design of RFIC components [11,12,13,14] or typical MEMS devices [15,16], such approaches have been overlooked for RF-MEMS devices which combine the two domains. For instance, an earlier work utilized metaheuristic optimization techniques in order to obtain optimal sets of design variables for planar CMOS RF inductors [13]. Similarly, in another work, a yield-aware multi-objective optimization strategy was introduced in order to optimize the accelerometer performance [16].

Undoubtedly, like any other conventional RF device, the performance of RF-MEMS devices largely depends on their geometry, and the design procedures may appear similar. However, their design process is certainly not the same and the optimization methods reported for conventional on-chip RF devices (optimized within a single domain, i.e., electromagnetic) cannot be directly applied to RF-MEMS-type passive components. Unlike conventional RF devices, RF-MEMS components exhibit a non-traditional structure and operation, along with a more involved design procedure spanning multiple physical domains, where mechanical, electrical and electromagnetic performance trade-offs need to be carefully optimized [17]. This intertwined design process spanning various physical domains induces a need to address the design optimization aspect for RF-MEMS devices. However, for RF-MEMS devices, there exist only a handful of attempts to address the design optimization issues [18,19,20]. Moreover, these efforts only focus on some specific RF applications (e.g., RF switches), and mostly restrict their discussions to a single physical domain [20]. Consequently, the current literature lacks in providing a generic multidisciplinary design optimization methodology for RF-MEMSs.

Accordingly, to bridge the current gap in RF-MEMS device design process, this work demonstrates a comprehensive design optimization methodology for monolithic RF-MEMS passive devices, using an automated multi-physics FEM (finite element method) framework coupled with heuristic multi-objective optimization techniques through machine-learning-based surrogate models. For a certain design space, the FEM framework generates a dataset describing the relation between input variables and corresponding device performance. Using this dataset, surrogate models are constructed that replace the extensive FEM simulations required in a typical iterative optimization process, thereby minimizing the computation cost overhead [11]. It is worthwhile to note that the computational cost of generating the dataset and corresponding surrogate models is essentially a one-time investment, and these surrogate models can be used repeatedly to quickly optimize various design scenarios while bypassing the time-consuming FEM simulations.

Like many other real-life engineering optimization problems, optimization of RF-MEMS devices is also a multi-objective problem, i.e., simultaneous optimization of many conflicting design objectives is needed. Likewise, a genetic algorithm-based multi-objective optimization algorithm (NSGA-II) is used in this work to optimize RF-MEMS devices [21]. The algorithm, NSGA-II, is capable of generating a set of optimal trade-offs (also called a pareto front or non-dominated solution set) between conflicting design objectives within a single run, which makes it an ideal candidate for solving multi-objective design problems, e.g., RF-MEMSs. Both FEM and optimization simulations are performed using commercially available software, i.e., COMSOL Multiphysics^®^ (COMSOL, Inc., Burlington, MA, USA) and MATLAB^®^ (MathWorks, Inc., Portola Valley, CA, USA). The remainder of this paper is organized into four different sections as follows: Section 2 elaborates on the complete optimization methodology; Section 3 and Section 4 present two optimization case studies, i.e., RF-MEMS inductors and electrostatic switches; and lastly, in Section 5, conclusions and future directions are given. To the best of the authors’ knowledge, this is the first work that illustrates a generic optimization strategy for on-chip RF-MEMS devices.

## 2. Proposed Multi-Objective Optimization Methodology

**Figure 1:**
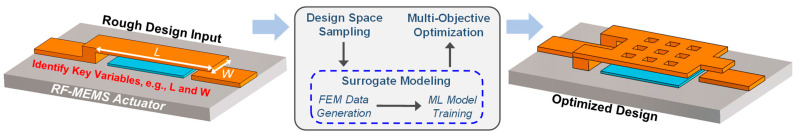
Schematic diagram demonstrating the generic design optimization methodology for RF-MEMS devices.

This section explains the proposed optimization methodology for RF-MEMS devices by providing details on the main steps involved in the process. A schematic illustration of the proposed optimization method is presented in Figure 1.

### 2.1. Problem Definition

The first step for the overall optimization approach is to formulate the target problem appropriately. In order to do so, critical performance parameters (design objectives), which need to be maximized or minimized for the intended device (e.g., capacitor, inductor, etc.) are identified. Next, the relevant design variables (which serve as inputs to the optimization problem) are defined within the feasible upper and lower bounds. Lastly, linear/non-linear design constraints and the fixed design parameters are added to the problem, which concludes the problem definition.

To elaborate further, consider the optimization problem of an RF-MEMS device labeled as ‘*A*’, where the design objectives to be optimized are given by a vector ***J_A_***. Meanwhile, the input variables are represented by ***x_A_***, and the design constraints and fixed parameters are ***G_A_*** and ***P_A_***, respectively. The design problem can then be mathematically expressed as:
*Design variables*:***x_A_*** = {*x*_*A*1_, ……, *x_An_*}*Optimize **J_A_***:***J_A_*** = {*J*_*A*1_, ……, *J_Am_*}*Subject to **G_A_*** ≤ *0*:***G_A_*** = {*G*_*A*1_, ……, *G_Ap_*}*Fixed parameters*:***P_A_*** = {*P*_*A*1_, ……, *P_Aq_*}where *n*, *m*, *p*, and *q* are the numbers of input design variables, design objectives, design constraints, and fixed parameters, respectively.

### 2.2. Finite Element Modeling Framework

In order to accurately model the electromechanical behavior of RF-MEMS devices, a sequentially coupled simulation routine, consisting of structural analysis and high-frequency analysis, is established. Corresponding to the values of the given design variables, first, structural modeling is performed, and the relevant results are recorded. Results from structural simulations are then fed to the high-frequency simulation setup, in which RF performance parameters are extracted. In this way, the effects of design variables on both structural and high-frequency performance parameters/objectives of the RF-MEMS devices are analyzed. The simulation setup is automated by means of MATLAB scripts to ensure automatic exploration of the whole design space, which is essential to locate the optimal points when FEM codes are subjected to optimization algorithms.

### 2.3. Surrogate Modeling

Ideally, for multi-objective design problems, FEM codes are directly linked with heuristic optimizers, where they are iteratively executed tens of thousands of times in search for an optimal solution space or a pareto front [11]. However, FEM simulations are computationally expensive, and hence drastically increase the time and computational costs for the overall optimization process. Alternatively, machine learning techniques can be used to map the FEM simulation data over the desired design space, into intelligent yet computationally cheap surrogate models capable of performing accurate predictions during optimization iterations. This technique, although less accurate, can drag down the computational costs into reasonable limits [11,12].

Similarly, in order to limit the computational costs, a machine-learning-enabled surrogate modeling approach is employed in this work. First, the design space chosen in the problem definition (i.e., range of design variables) is sampled. To effectively explore the whole design space, the Latin Hypercube sampling technique is chosen. For this work, the sampling size is restricted to 5*^n^*, where *n* corresponds to the number of design variables present in the problem. It should be noted that the sampling size of 5*^n^* has been chosen carefully to minimize the time required for data generation with FEM simulations, while efficiently covering the whole design space. However, the number of samples can be increased if one needs to cover a complex design space. Next, FEM simulations are carried out for the entire sampled design space and corresponding values of the design objectives are recorded.

Thereafter, this FEM-driven dataset is utilized to train the decision-tree-based regression models, using the “Statistics and Machine Learning Toolbox” of MATLAB^®^ [22], which directly reflect the input–output relationship between design variables and objectives. Initially, various model presets (including support vector machines, regression trees, and linear regression models) offered by the MATLAB ‘Regression Learner’ module are used to fit the data generated through FEM simulations. Among them, regression trees are selected for this work, since they have high interpretability, low computational cost, high prediction accuracy, and can efficiently capture non-linear relationships in data [22,23,24,25]. However, it must be noted that no ML model can serve as the “universal” model or fit to all types of datasets, and therefore the optimal model type may vary among different datasets. For our dataset, we observed that regression trees offer the fastest training time and highest accuracy among all compared models.

Regression trees are a variant of decision trees, which fit real-valued numerical data [26]. Regression trees work on the principle of binary recursive splitting, which corresponds to an iterative process of successively splitting data into two branches, mimicking a tree-like structure, by which the split location is decided based on the mean square error (“split rule”: data is split at the location which results in the minimum mean squared error). In particular, the algorithm starts by grouping all the data records in one partition, which is called the “root node”. Next, the splitting process starts and continues along each branch, until the partition size falls below a certain threshold, or the mean squared error within the partition falls to zero, representing the “leaf nodes”. Moreover, a *k*-fold validation scheme is adopted during the training of regression tree models, where *k* = 5. The accuracy of these metamodels is ensured by tracking commonly used accuracy metrics, including root mean square error (RMSE), relative RMSE, and coefficient of determination (*R*^2^).

### 2.4. Multi-Objective Optimization

The trained surrogate models, along with the information on design variables and constraints, are then subjected to a multi-objective optimizer that reveals pareto optimal points for conflicting design objectives. In the proposed approach, a variant of the non-dominated sorting genetic algorithm (NSGA-II) is utilized to solve the RF-MEMS optimization problem [21]. The terms non-dominated solution set and pareto front are interchangeably used, and defined as the points at which no further improvement in one objective is possible without degrading at least one of the other objectives.

**Figure 2:**
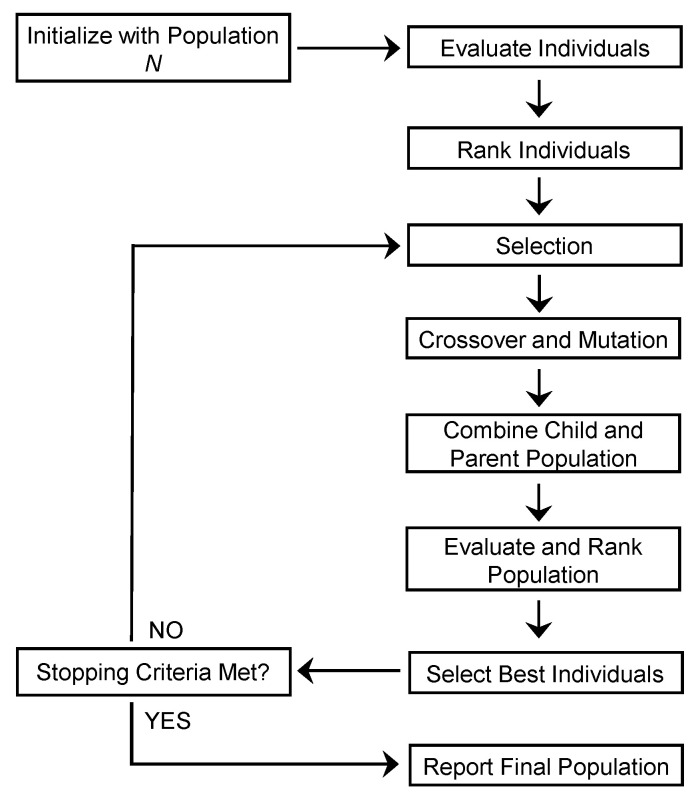
NSGA-II algorithm flowchart [21].

The algorithm starts with an initial design variable population of size *N*. In order to ensure a thorough exploration of the whole design space, the population size was set to 200 in this work. Next, the initial population is evaluated to obtain the respective objective function values (fitness values). The evaluated population is then ranked based on the fitness values, and the best individuals among the initial population are selected as a parent population. In this work, the best half (50%) of the whole population is selected as the parent population, while the other half is discarded. By means of genetic operators (crossover and mutation), a child population is then created from the parent population and both child/parent populations are merged to make a single extended population. For this work, the crossover fraction and mutation rate are set to 0.8 and 0.05, respectively. At this point, all the individuals of the extended population are evaluated in order to obtain the corresponding objective functions’ values associated with them. Lastly, the individuals of the extended population are ranked and sorted on the basis of their non-dominance level. The best individuals from the sorted individual sets are selected as the parent population for the next iteration and fed back to genetic operators. This iterative cycle continues until the stopping criteria is met, i.e., when the average relative difference between non-dominated sets of two consecutive iterations falls below a certain value (1 × 10^−4^ is used as a threshold value). A complete flowchart of the NSGA-II algorithm can be viewed in Figure 2.

## 3. Case Study: RF-MEMS Suspended Inductor Optimization

On-chip inductors are widely used in many RF transceiver blocks, such as low noise amplifiers, voltage-controlled oscillators, DC–DC converters, matching circuits, passive filters, etc. [2]. Critical performance parameters for monolithic inductors are inductance (*L*), *Q*-factor (*Q*), and required footprint area. Conventional planar spiral geometries of RF inductors lying on a semiconductor substrate suffer from large substrate losses, and hence offer low *Q*-factor values [2]. Alternatively, in order to minimize substrate proximity losses, MEMS technologies offer many techniques to develop out-of-plane RF inductors with improved *Q*-factors. Some common techniques for developing 3D high-Q on-chip inductors are plastic deformation magnetic assembly to achieve vertical inductors [27], thick photoresist processing to fabricate elevated suspended inductors [28], and stress-induced self-assembled thin film inductors [2], etc.

For this case study, MEMS-based stress-induced self-assembled on-chip loop inductors were considered to demonstrate the functionality of the proposed design optimization methodology. In order to maximize inductor performance while minimizing the footprint area, three design objectives were chosen, i.e., *Q*-factor, inductance (nH), and area (mm^2^). Furthermore, the geometrical parameters of inductor coil, i.e., radius (*r*), conductor width (*w*), and metal thickness (*t*), as well as the operating frequency (*f*), were taken as design variables (Table 1).

**Figure 3:**
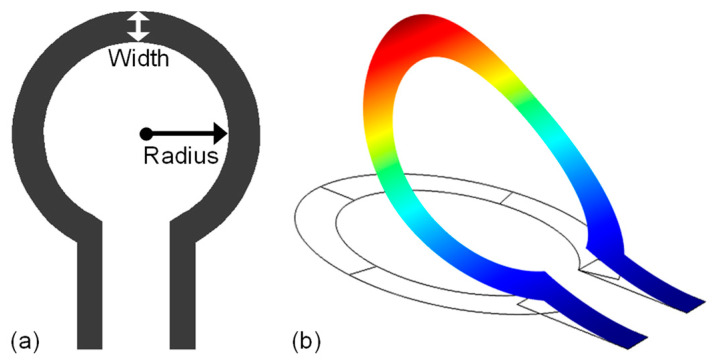
Self-assembled RF-MEMS ring inductor: (**a**) Top view of the planar ring inductor geometry prior to out-of-plane self-assembly; and (**b**) post self-assembly view of the deformed ring inductor.

Although RF-MEMS inductors offer excellent RF performance, their mechanical reliability is limited, primarily due to their suspended structure [29], which inherently complicates the design process and makes manual design optimization even more challenging. In order to address this issue in our case study, we constrained the maximum stress value (*S*) in the inductor structure below the yield strength of the material (205 MPa for gold, which is used as a structural material in simulations), to ensure a mechanically reliable design. A simplified 2D top view of the inductor layout is provided in Figure 3a.

### 3.1. Finite Element Modeling of RF-MEMS Loop Inductors

#### 3.1.1. Mechanical Analysis

Stress-induced self-assembly of patterned thin films into out-of-plane loop inductors was studied using a static structural analysis, in which the self-assembly process was artificially replicated using quasi-static FEM simulations [30]. Modeling results for the structural deformation of a typical ring inductor are shown in in Figure 3b.

#### 3.1.2. High-Frequency Analysis

The deformed inductor geometries obtained from mechanical analysis were then imported to the next FEM code, where high-frequency AC analysis was performed. The inductors were treated as two-port RF networks and relevant Z-parameters were extracted at the target frequencies. The Z-parameters for inductors were then converted to *Q*-factor and inductance values through the following relationships:

(1) $$Q=\frac{Im(Z_{11})}{Re(Z_{11})}$$

(2) $$L=\frac{Im(Z_{11})}{\omega }$$

### 3.2. Multi-Objective Optimization

Prior to solving the optimization problem, the defined design space for inductors was sampled with a sampling size of 5^3^(125), in order to fully cover the entire design space. Next, the FEM code (containing mechanical and EM simulations, as described above) was run in an automated loop for all 125 samples in order to acquire the corresponding values for design objectives (*Q*, *L*, and area), which took 31 h using an Intel core i5 computer. This FEM data was then used to train the regression trees, where the training time was 0.5 s. The accuracy of the developed metamodels of inductance and *Q*-factor was validated using the metrics listed in Table 2.

In order to comprehensively model the trade-offs between the performance parameters of MEMS inductors, various combinations of design objectives were exploited. Initially, only two design objectives (inductance and area) were considered to trace the optimal inductance density points at a frequency of 5 GHz. In this particular case, the inductor optimization problem can be mathematically expressed as:
*Design variables*:***x*** = {*r*, *w*, *t*}*Optimize **J***:***J*** = {*L*, *area*}*Subject to **G***:***G*** = {*Q* ≥ *10*, *S* ≤ *205* MPa}*Fixed parameters*:***P*** = {*f* = *5* GHz}

**Figure 4:**
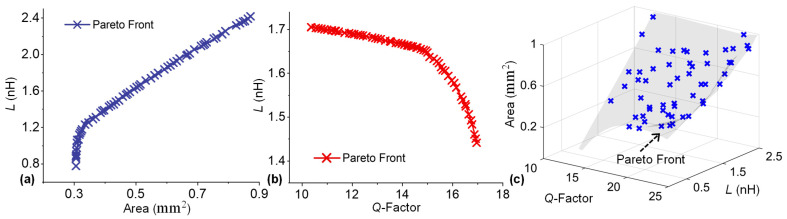
Non-dominated solution sets for RF-MEMS inductor optimization problem: (**a**) inductance vs. area; (**b**) *Q*-factor vs. inductance; and (**c**) *Q*-factor vs. inductance vs. area.

With these conditions, the developed surrogate models were subjected to the optimizer, and the extracted non-dominated solution set for inductance and footprint area is presented in Figure 4a. Interestingly, the coil’s inductance and footprint area exhibit strong conflicting natures; in other words, the inductance of a coil is directly proportional to the coil diameter, whereas the footprint area increases with the increasing diameter [31]. A similar trend was observed for the inductance-area pareto plot (Figure 4a), where the inductance first increased sharply with increasing area. However, for an area above 0.3 mm^2^, the inductance followed a near-linear trend.

Another noticeable trade-off in the inductor design process is displayed by the correlation between the inductor’s quality factor and inductance. For instance, an increased coil diameter promises an increased coil inductance, but on the other hand, simultaneously reduces the coil *Q*-factor, due to increased series resistance [32]. Likewise, for the selected design space, a search for pareto front between the inductor’s inductance and *Q*-factor was performed, indicating the below formulated optimization problem.
*Design variables*:***x*** = {*r*, *w*, *t*}*Optimize **J***:***J*** = {*Q*, *L*}*Subject to **G***:***G*** = {*Area* ≤ *1* mm^2^, *S* ≤ *205* MPa}*Fixed parameters*:***P*** = {*f* = *5* GHz}

The obtained pareto front plotted in Figure 4b illustrates the complex conflicting relationship between the *Q*-factor and inductance, where inductance initially decreases slowly with an increase in the *Q*-factor, and then suddenly starts to drop near a *Q*-factor of 15.

In order to demonstrate the applicability of proposed methodology to a higher number of design objectives, a search for pareto optimal points among all three design objectives (*Q*, *L,* and area) was performed, which revealed a three-dimensional pareto front, shown in Figure 4c. The optimization problem in such case can be expressed as:
*Design variables*:***x*** = {*r*, *w*, *t*}*Optimize **J***:***J*** = {*Area*, *Q*, *L*}*Subject to **G***:***G*** = {*S* ≤ *205* MPa}*Fixed parameters*:***P*** = {*f* = *5* GHz}

**Figure 5:**
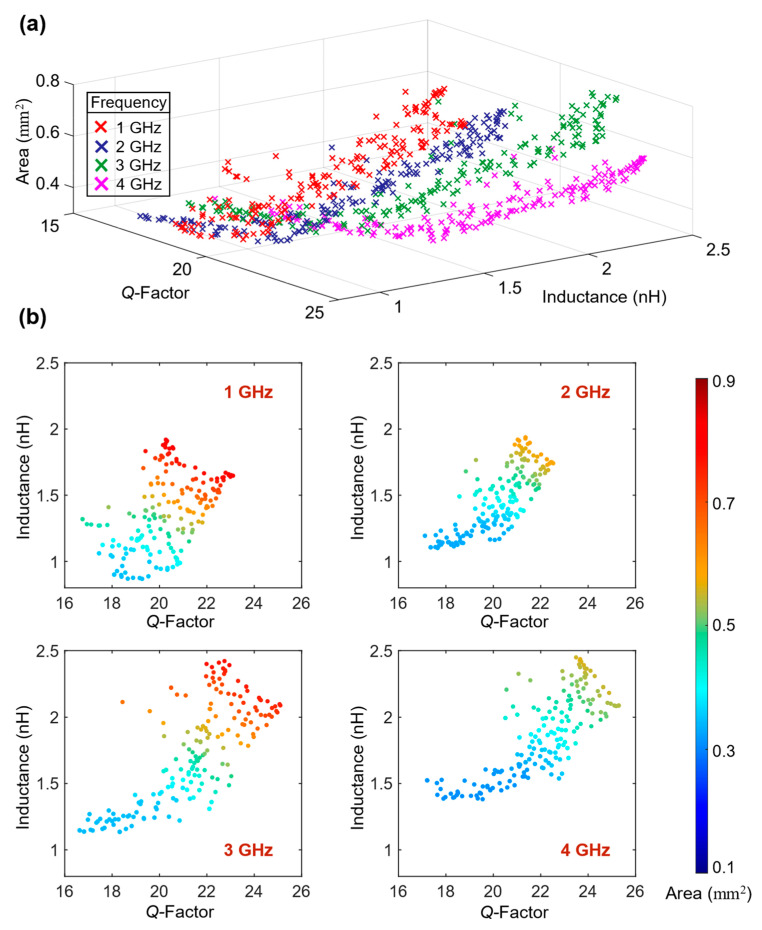
Pareto fronts for RF-MEMS inductor at four different frequencies between 1 and 5 GHz: (**a**) combined pareto plot for all frequencies; and (**b**) individual pareto plots at different frequencies.

In the above-described optimization scenarios, the frequency was fixed to 5 GHz. Nevertheless, the proposed methodology can easily be tailored to achieve the broadband optimization of RF-MEMS devices. Since the frequency is taken as an input variable in this case study (Table 1), the developed surrogate models essentially describe the frequency-dependent input–output behavior of RF-MEMS inductors. That is to say, following the development of frequency-dependent surrogate models, the inductors can be quickly optimized for various operating frequencies by directly using these frequency-dependent surrogate models. To further illustrate this idea, we optimized the inductor surrogate models at four different frequencies (1, 2, 3 and 4 GHz) and extracted the corresponding pareto optimal points, as plotted in Figure 5. These intrinsic trade-off curves, related to the inductor geometry and relevant performance parameters, are difficult to extract and optimize manually. Hence, information on the optimality of such trade-offs is highly valuable to RF designers.

## 4. Case Study: RF-MEMS Switch Optimization

MEMS switches play a key role in many RF circuitries for routing signals through transmission lines. Compared to their active counterparts (i.e., transistor switches), MEMS switches are highly linear, and offer lower ON-state losses and higher OFF-state isolation over a wide bandwidth [6]. Among many actuation principles, electrostatic actuation is the most commonly used in RF switches because of its simple operation and ease of integration with standard IC fabrication processes [33,34]. Correspondingly, cantilever-based series RF-MEMS switches were selected as a second demonstrator, to further highlight the significance and validate the adaptability of the formal design optimization method reported herein.

**Figure 6:**
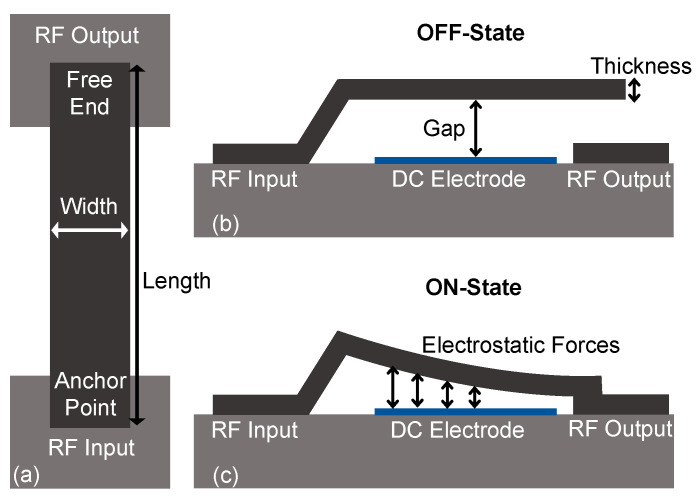
Cantilever-based series RF-MEMS switch: (**a**) top view; (**b**) side view in OFF-state; and (**c**) side view in ON-state.

Electrostatic RF-MEMS switches are characterized by three major performance parameters, including the actuation voltage or pull-in voltage (*V_P_*), ON-state insertion loss (*S_21-ON_*), and OFF-state signal isolation (*S_21-OFF_*), which were considered as the design objectives for this study. Since RF-MEMS switches indicate a complex electromechanical system and are prone to reliability issues such as stiction [35], this case study also considers two highly critical reliability-related design constraints, i.e., maximum stress at anchors during actuation (*S*) and beam stiffness (*k*), thereby demonstrating a more practical optimization route via the proposed optimization methodology. Moreover, the design variables considered for an electrostatic series switch were length (*l*), width (*w*), thickness (*t*) of the cantilever beam, and the air gap (*g*) between the beam and the underlying actuation electrode. Lower and upper limits on the values that can be assumed by the target design variables are summarized in Table 3, while the topology of the series MEMS switch subject to the optimization workflow described in this work is shown in Figure 6.

### 4.1. FEM Modeling of RF-MEMS Switches

#### 4.1.1. Pull-In Analysis

To extract information on actuation voltages, MEMS switches are first modeled as electromechanical structures representing a multi-physics problem. Following the definition of necessary boundary conditions, the voltage between the cantilever switch and underlying DC electrode is increased gradually, which induces a proportional deflection in the fixed-free cantilever switch. At this point, the electrostatic forces are balanced by the elastic restoring force of the cantilever. However, with increasing voltage, when the electrostatic forces outrun the spring-restoring forces, the system becomes unstable, and the moveable cantilever beam collapses down to the fixed DC electrode (pull-in point, Figure 6c) [31].

From FEA perspective, no stable static solution exists for electrostatic actuators at voltage values greater than or equal to the pull-in voltage, and hence the solution diverges. Consequently, locating the exact pull-in value directly is not possible with FEM solvers, and solution divergence is usually treated as an indicator to estimate the pull-in voltage [36]. Thus, in order to achieve a more precise estimation of the pull-in voltages for specified MEMS switches in an automatic fashion, an iterative algorithm was established that could locate the pull-in voltage values with a maximum error of less than 0.1 V. The key steps for the automatic detection of pull-in voltage are listed below:

1. Apply the initial voltage *V*_0_ = 0 volts.
2. Solve for electromechanical forces. If the solution converges, go to step 3, else reduce the increment voltage *V*_1_ by half, i.e., *V*_1_ = 0.5 *V*_1_, to gradually converge to a solution, and then go to step 4.
3. Increase the applied voltage with an increment of *V*_1_ (volts), i.e., *V*_0_ = *V*_0_ + *V*_1_. Go back to step 2.
4. If *V*_1_ < 0.05 V (tolerance for stopping criteria), go to step 7, else decrease the applied voltage, i.e., *V*_0_ = *V*_0_ − *V*_1_, and go to the next step.
5. Solve for electromechanical forces. Replace *V*_1_ = 0.5 *V*_1_. If the solution converges, go to step 6, else go back to step 4.
6. If *V*_1_ < 0.05 V (tolerance for stopping criteria), go to step 7, else increase the applied voltage with an increment of *V*_1_ (volts), i.e., *V*_0_ = *V*_0_ + *V*_1_. Go back to step 5.
7. Stop the code. Pull-in voltage (*V_P_*) = current value of *V*_0_.

#### 4.1.2. High-Frequency Analysis

Once the pull-in study was concluded, switch geometries for both the ON-state and OFF-state were analyzed for their high-frequency responses. Considering the sizeable contribution of MEMS switches for mm-wave band circuits, the operating frequency for this study was fixed at 28 GHz. The high-frequency models for switches were solved for two-port network s-parameters, specifically *S*_21_, which represents the insertion losses and signal isolation for the switch in the ON-state and OFF-state, respectively.

### 4.2. Multi-Objective Optimization

Initially, the design space for RF-MEMS switches was sampled into 5^4^ (625) samples and the corresponding values for design objectives (insertion loss, isolation, and actuation voltage) were obtained using an automated FEM code, accounting for approximately 104 h of computation time on an Intel core i5 computer. Subsequently, this dataset, of size 625, was used for regression model training, with a training time of ~1.6 s. The accuracy of trained metamodels for the objective functions of an RF-MEMS switch is illustrated in Table 4.

At this stage, the equivalent surrogate models acquired for the design objectives of the switch were subjected to the optimizer, and multi-objective optimization was performed. Essentially, the effects of geometrical parameters of the switch on the corresponding RF isolation and actuation voltage values are contradictory to each other, which creates a non-trivial trade-off in electrostatic MEMS switch design process [3]. For example, an increased air gap between the switch beam and the underlying electrode improves the RF isolation, but at the cost of higher actuation voltage [3]. Accordingly, we first identified the pareto optimal points for RF isolation and actuation voltage for the considered electrostatic series MEMS switch using a non-dominated sorting algorithm, and this optimization scenario can be formulated as:
*Design variables*:***x*** = {*l*, *w*, *t*, *g}**Optimize **J***:***J*** = {*V_P_*, *S*_21-*OFF*_}*Subject to **G***:***G*** = {*k ≥ 10* N/m, *S ≤ 205* MPa}*Fixed parameters*:***P*** = {*f* = *28* GHz}

**Figure 7:**
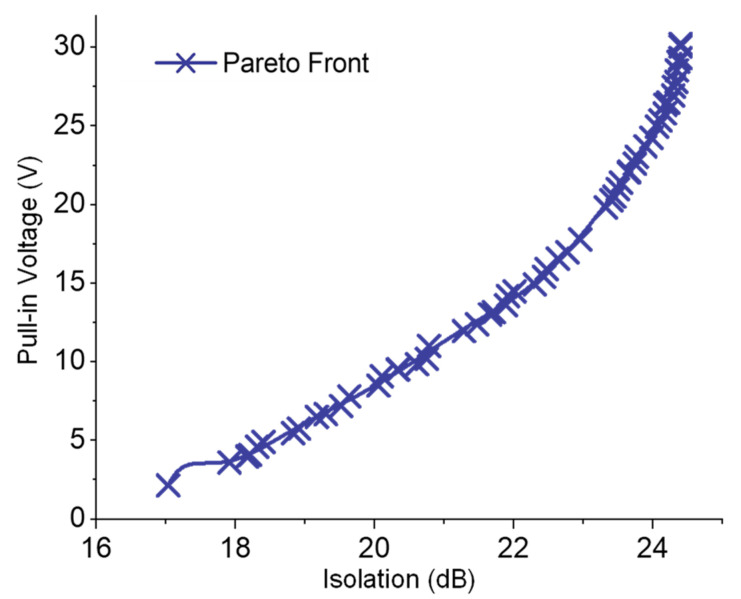
Set of optimal trade-offs between the RF isolation and pull-in voltage of the cantilever-based series RF-MEMS switch.

Notably, in order to ensure a mechanically robust design, the maximum stress at the switch anchors was constrained below the yield strength of the switch material (gold). Meanwhile, the beam stiffness was restricted to above 10 N/m, as suggested in [35]. The extracted optimal solution set shown in Figure 7 validates the contrasting behavior of pull-in voltage and RF isolation, where, for the chosen design space, the optimal isolation and pull-in values vary from 3 V to 30 V and from 15.5 dB to 24.2 dB, respectively. Furthermore, identification of these variations in an optimal solution set enables designers to explore the design space effectively.

**Figure 8:**
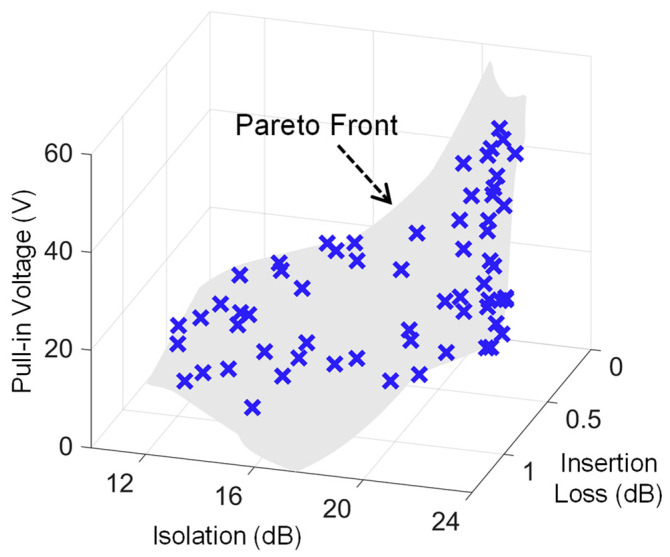
Three-dimensional pareto front demonstrating trade-off among actuation voltage, RF isolation, and insertion losses for the RF-MEMS series switch.

Lastly, in order to fully optimize the overall performance of the switch, a multi-objective pareto search with all the design objectives was carried out, and the corresponding three-dimensional pareto front shown in Figure 8 was obtained. The relevant mathematical formulation for this optimization case is given as:
*Design variables*:***x*** = {*l*, *w*, *t*, *g*}*Optimize **J***:***J*** = {*V_P_*, *S*_21-*ON*_, *S*_21-*OFF*_}*Subject to **G***:***G*** = {*k ≥ 10* N/m, *S ≤ 205* MPa}*Fixed parameters*:***P***
= {*f = 28* GHz}


For a fixed design space, the 3D plot comprehensively displays the relationships among various design objectives, and can be used as a tool to handle various design scenarios. For instance, considering a design problem in which low pull-in voltage is the prime requirement, a pareto optimal design with the lowest pull-in voltage can be readily identified and selected from the plot.

## 5. Conclusions

While recent studies attempt to satisfy the needs for RF-MEMS devices, mainly by adding more efficient novel designs into the literature, the discussions on design optimization division are somewhat limited. The design optimization aspect, if modeled properly, not only improves the performance of any particular RF-MEMS device, but also provides a better insight into the device’s behavior for the RF designers. Accordingly, this study aims to highlight the significance of design optimization for RF-MEMS devices by presenting a machine-learning-based generic optimization approach. The overall optimization process is flexible and can easily be customized to fit any RF-MEMS device. The proposed strategy is validated for two common RF-MEMS devices (inductors and switches) using the NSGA-II multi-objective optimization algorithm, and corresponding results in the form of optimal design trade-offs (pareto fronts) are reported. The accuracy of the reported results can be further verified with physical device realizations in future studies.

## Figures and Tables

**Table 1:** Design variables for RF-MEMS inductor.

Design Variables	Lower Bound	Upper Bound
Thickness (*t*)	1 µm	5 µm
Coil radius (*r*)	300 µm	600 µm
Width (*w*)	60 µm	100 µm
Frequency (*f*)	1 GHz	5 GHz

**Table 2:** Accuracy metrics for meta-models of *L* and *Q*.

Objective	R^2^	RMSE	Relative RMSE
Inductance (*L*)	0.91	0.14	8%
*Q*-factor (*Q*)	0.92	0.75	4%

**Table 3:** Design variables for RF-MEMS switch.

Design Variables	Lower Bound	Upper Bound
Thickness (*t*)	1 µm	5 µm
Gap (*g*)	2 µm	8 µm
Width (*w*)	50 µm	80 µm
Length (*l*)	300 µm	600 µm

**Table 4:** Accuracy of meta-models of switch’s objective functions.

Objective	R^2^	RMSE	Relative RMSE
Pull-in voltage	0.94	0.69	5%
Insertion Loss	0.96	0.09	2%
Isolation	0.90	0.78	7%

## Data Availability

Data available on request from the corresponding author.
